# Family functioning and dysfunctional attitudes mediate the link between childhood maltreatment and nonsuicidal self-injury in depressed college students: a moderated mediation model of anhedonia

**DOI:** 10.3389/fpsyt.2025.1649915

**Published:** 2025-10-01

**Authors:** Huawei Tan, Fan Zhang, Xiaofen Zong, Gui Gui, Hanping Bai, Huiling Wang, Shenhong Weng, Zhongchun Liu

**Affiliations:** ^1^ Department of Psychiatry, Renmin Hospital of Wuhan University, Wuhan, Hubei, China; ^2^ Department of Orthopedics, Renmin Hospital of Wuhan University, Wuhan, Hubei, China; ^3^ Taikang Center for Life and Medical Sciences, Wuhan University, Wuhan, Hubei, China

**Keywords:** childhood maltreatment, nonsuicidal self-injury, major depressive disorder, family functioning, dysfunctional attitudes, anhedonia, cognitive vulnerability model

## Abstract

**Objective:**

Childhood maltreatment is a well-established risk factor for nonsuicidal self-injury (NSSI), particularly in individuals with major depressive disorder (MDD). The psychological mechanisms are complex and not completely understood. Based on the Cognitive−Behavioral Vulnerability Model, this study aimed to investigate whether family functioning and dysfunctional attitudes act as parallel mediators of the relationship between childhood maltreatment and NSSI and anhedonia moderates the association between dysfunctional attitudes and NSSI in depressed college students.

**Methods:**

525 college students diagnosed with MDD were recruited from the ESCID project. They completed Childhood Trauma Questionnaire (CTQ), Family Assessment Device (FAD), Dysfunctional Attitude Scale (DAS), Snaith-Hamilton Pleasure Scale (SHAPS), and a measure of NSSI. Data were analyzed using SPSS 26.0 for descriptive statistics and correlations, and Mplus 8.3 for mediation and moderated mediation analyses, employing maximum-likelihood estimation with Monte Carlo integration.

**Results:**

Childhood maltreatment showed a positive correlation with dysfunctional attitudes, anhedonia, and NSSI, while family functioning demonstrated no significant association. Mediation analyses revealed that childhood maltreatment had both a direct effect on NSSI (β = 0.125, *p* < 0.05, 95% CI [0.010, 0.240]) and an indirect effect through dysfunctional attitudes (β = 0.035, *p* < 0.05, 95% CI [0.008, 0.063]). In contrast, the mediating role of family functioning was not significant (β = −0.060, *p* > 0.05), as was the total indirect effect. Moderated mediation analyses showed that anhedonia significantly moderated the association between dysfunctional attitudes and NSSI (β = 0.190, *p* < 0.001, 95% CI [0.092, 0.288]). Simple slope tests indicated that dysfunctional attitudes exhibited a stronger correlation with NSSI in individuals with high levels of anhedonia, while this correlation became nonsignificant at lower levels.

**Conclusions:**

Our findings reveal that dysfunctional attitudes significantly mediate the link between childhood maltreatment and NSSI, while family functioning showed no mediating effect. Anhedonia amplified the impact of dysfunctional attitudes on NSSI, suggesting that cognitive and affective vulnerabilities enhance the risk of self-injury. Interventions that target dysfunctional attitudes and enhance hedonic capacity may improve prevention and treatment among maltreated youth with depression.

## 1 Introduction

Major depressive disorder (MDD) is a prevalent mental health condition worldwide, recognized as a primary contributor to disability and disease burden. Global estimates indicate that around 290 million individuals were affected by depression in 2019, demonstrating a consistent rise in its prevalence (1, 2). Notably, the prevalence of depressive symptoms or diagnosed MDD is much higher among college students than in the general adult population. A large-scale survey conducted in the post-pandemic era reported that 48.9% of college students exhibited depressive tendencies (3).

Nonsuicidal self-injury (NSSI) is defined as deliberate and repeated self-harm without the intention of suicide (4). Common methods include cutting, burning, scratching, and self-hitting (5). This behavior is highly prevalent among college students, particularly those diagnosed with MDD (6). A multinational study of college students reported a lifetime prevalence of NSSI at 17.7%, and a 12-month prevalence of 8.4% (7). In China, a systematic review reported that lifetime NSSI prevalence among college students is about 21.2%, higher than many international estimates, with male students at approximately 24.5% and female students at about 20.4% (8). Studies have found that approximately 34.2% of depressed college students are involved with NSSI, a rate notably greater than that observed in non-depressed students (9), which underscores the urgent need to identify its risk factors. Among this population, NSSI not only serves as a robust predictor of future suicide attempts (10), but is also linked to greater depressive symptom severity, higher rates of suicidal ideation and attempts, and poorer treatment outcomes (11). These findings highlight the urgent need to clarify the risk factors of NSSI in depressed college students to inform prevention and early intervention.

Childhood maltreatment, which includes physical, sexual, and emotional abuse and neglect, is one of the main risk factors for NSSI (12). Prior research indicates that college students with MDD and a history of childhood maltreatment tend to report higher levels of depressive symptoms and a greater risk of NSSI (13). Furthermore, recent researches indicate that NSSI, depressive symptoms, and difficulties with emotion regulation are linked to childhood maltreatment (14, 15). Recent evidence indicates that childhood maltreatment (CM) is associated not only with the occurrence of NSSI but also with its frequency, as college students with experiences of emotional or sexual abuse are more likely to engage in repetitive and persistent self-injury (16). The mediating and moderating roles in the pathway from childhood maltreatment to NSSI are complicated and still unknown. To elucidate the mechanisms relating to childhood abuse and NSSI, multivariate models that evaluate both mediators and moderators are required.

Family functioning represents a family’s capacity to maintain stability through effective communication, problem-solving, and role regulation (17). About half of first-year students report childhood household dysfunction, such as parental mental illness or domestic violence (18). Childhood maltreatment, including abuse and neglect, is a known risk factor for both depression and impaired family functioning (19). It undermines communication and cohesion, increasing conflict and weakening resilience (20, 21). Poor family functioning, separation, and insufficient parental support are linked to emotional dysregulation and social isolation, elevating NSSI risk (22). Prior studies have consistently identified family functioning as a predictor of NSSI (23, 24). Taken together, these findings suggest that family functioning may serve as a mediating mechanism linking childhood maltreatment to NSSI. However, existing research indicates that family dysfunction may mediate the link between childhood adversity and NSSI in adolescents (25); its specific role in linking childhood maltreatment to NSSI remains underexplored, particularly in clinical populations such as college students with MDD.

Dysfunctional attitudes (DA) are one of the main cognitive susceptibility factors in depression, which are defined by inflexible, absolutist, and negatively biased ideas about oneself, other people, and the future. These maladaptive cognitive schemas contribute to the development and maintenance of depressive symptomatology and frequently show up as hopelessness, excessive self-criticism, and perfectionism (26). The development and persistence of NSSI are further influenced by DA, particularly when individuals internalize negative beliefs that lead to a decline in self-worth and an increase in emotional distress (27). According to empirical research, dysfunctional attitudes increase negative affect and impair adaptive coping strategies, which in turn increase sensitivity to NSSI. It makes NSSI seem like a reasonable measure to deal with bad feelings in a bad way (28). Additionally, DA may facilitate the connection between childhood maltreatment and NSSI since early traumatic experiences foster the development of inflexible negative schemas, therefore heightening the propensity for self-injury (29).

Anhedonia is characterized by the incapacity to derive pleasure or satisfaction from activities that were once enjoyable, including eating, exercising, or socializing (30). It is a fundamental symptom of MDD and a distinguishing trait of numerous psychological and physical health issues (31). Anhedonia is particularly critical in teenagers due to its association with more severe clinical consequences, including an increased risk of chronicity and suicidality (32). Recent empirical research indicates that adolescents with significant anhedonia demonstrate compromised reward processing, diminished emotional reactions, and increased susceptibility to maladaptive coping mechanisms, including NSSI (33). In recent years, researchers have increasingly examined the appearance and effects of anhedonia among college students. Anhedonia frequently coincides with cognitive impairment and heightened stress sensitivity in students with MDD, hence increasing the likelihood of self-injurious behavior (34). Patients with depression frequently exhibit cognitive biases, including an increased focus on negative information, which diminishes their capacity to enjoy pleasure from happy events. Empirical research indicates that anhedonia substantially hinders emotional control in adolescents, thereby elevating the probability of maladaptive behaviors (35).

The Cognitive–Behavioral Vulnerability Model (CBVM) provides an integrative framework for comprehending the development of maladaptive behaviors (36). It posits that early adverse experiences can foster lasting vulnerabilities that operate through distinct yet interconnected mechanisms. These mechanisms include environmental pathways, such as disruptions in family or social contexts, and cognitive pathways, such as the development of rigid and maladaptive belief systems. Moreover, emotional vulnerabilities—especially deficiencies in experiencing or maintaining positive affect—may interact with these mechanisms, undermining protective processes and increasing risk. These vulnerabilities collectively heighten the probability of maladaptive outcomes such as NSSI (37).

**Figure 1:**
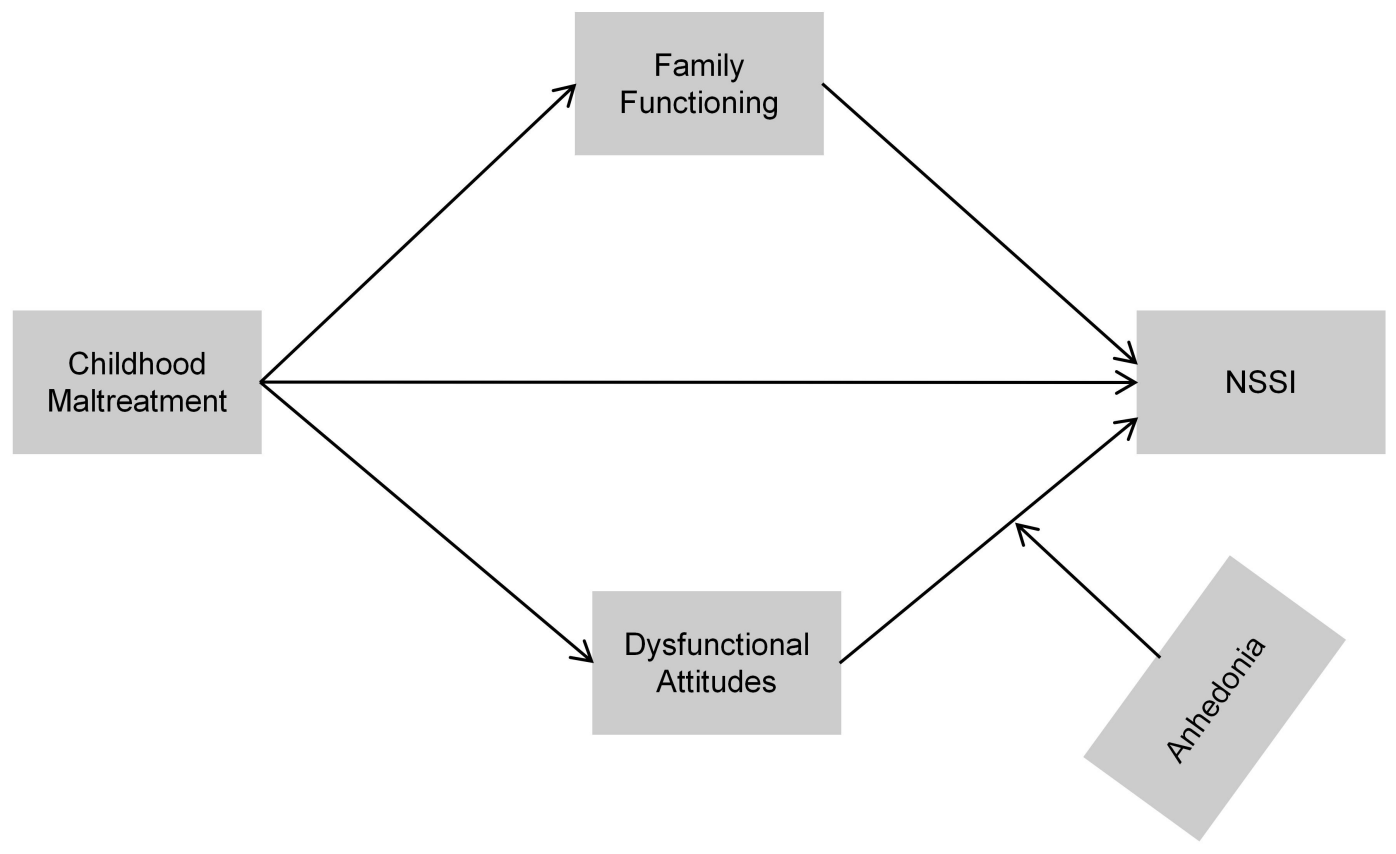
Hypothesized conceptual model illustrating the proposed mediation and moderation pathways linking childhood maltreatment to NSSI.

Utilizing this framework, the present study conceptualizes childhood maltreatment as a distal adverse experience that may generate both environmental and cognitive vulnerabilities. On the environmental side, childhood maltreatment is expected to compromise family functioning, as evidenced by decreased support, communication, and cohesion. On the cognitive side, it is expected to cultivate rigid, self-critical, dysfunctional attitudes. Furthermore, anhedonia is regarded as a typical emotional vulnerability that may amplify the impact of dysfunctional attitudes on NSSI by weakening the protective influence of positive experiences. Based on this theoretical rationale, we propose an integrative model in which childhood maltreatment predicts NSSI through the parallel mediation of family functioning and dysfunctional attitudes, with anhedonia moderating the effect of dysfunctional attitudes on NSSI. The conceptual framework is presented in **Figure 1**. Accordingly, the study evaluates the subsequent hypotheses:

Hypothesis 1: Dysfunctional attitudes serve as a mediator in the association between childhood maltreatment and NSSI.
Hypothesis 2: Family functioning serves as a mediator in the link between childhood maltreatment and NSSI.
Hypothesis 3: Anhedonia moderates the relationship between dysfunctional attitudes and NSSI.

This study sought to provide a comprehensive model of the cognitive, affective, and behavioral mechanisms related to NSSI among college students with MDD, while also informing early intervention strategies through the analysis of the interactions among these mechanisms.

## 2 Methods

### 2.1 Setting and participants

This cross-sectional study was conducted from June 2019 to November 2020 as part of the Early-Warning System and Comprehensive Intervention for Depression (ESCID), a large-scale program on the etiology, relapse mechanisms, and psychosocial interventions of MDD (38). Participants were recruited from outpatient clinics in the Department of Psychiatry at Renmin Hospital of Wuhan University, outpatient clinics at collaborating hospitals, and college counseling centers. A total of 1,865 patients with MDD were initially screened. After applying the predefined inclusion and exclusion criteria, 1,340 individuals were excluded, and the final analytic sample comprised 525 college students with clinically confirmed MDD.

Inclusion criteria were (1): age 18–28 years (2); current enrollment as a college or postgraduate student (3); MDD diagnosis independently confirmed by two psychiatrists according to DSM-5 (39) (4); verification of a current episode (ongoing or within two weeks) with the Mini-International Neuropsychiatric Interview (MINI) (40) (5); ability to complete assessments in Chinese, provide informed consent, attend visits or online follow-up, and have smartphone/internet access. Exclusion criteria were (1): schizophrenia or bipolar disorder (2); severe cognitive or language impairments (3); pregnancy or lactation (4); current or past substance/alcohol abuse; and (5) participation in other clinical trials.

Self-report questionnaires were administered via electronic tablets, and clinician-rated instruments were completed by trained research assistants to guarantee data integrity. Assessments required approximately 15–20 minutes. The study was approved by the Ethics Committee of Renmin Hospital of Wuhan University (No. WDRY2020-K191) and conducted in accordance with the Declaration of Helsinki. Written informed consent was obtained from all participants, who were informed of their right to withdraw at any time without penalty.

### 2.2 Measurements

#### 2.2.1 Compilation of general information

Demographic and clinical characteristics such as age, gender, and first-episode MDD were obtained by a structured self-report questionnaire.

#### 2.2.2 Assessment of nonsuicidal self-injury

Nonsuicidal self-injury (NSSI) was assessed using the Ottawa Self-Injury Inventory (OSI), a widely used self-report tool for evaluating self-injurious behavior (41). The OSI has demonstrated good reliability and validity in samples of Chinese college students (42). In the present study, we focused on two core items of the OSI to classify NSSI status: whether the individual had engaged in deliberate self-injury without suicidal intent in the preceding 12 months and the method of self-injury employed. According to these criteria, participants were divided into two groups: the non-NSSI group (coded as 0) included those who had not engaged in NSSI behavior in the prior year (0 times), and the NSSI group (coded as 1) included those who had experienced at least one episode (≥1 time). This categorization method has been extensively utilized in prior research (43, 44).

#### 2.2.3 Childhood maltreatment

The Childhood Trauma Questionnaire-Short Form (CTQ-SF), created by Bernstein et al., is a commonly used self-report tool for retrospectively assessing abuse and neglect experiences prior to the age of 16 years (45). There are 28 items on the scale, 25 of which are clinical and 3 of which are for validity. There are five subscales for the 25 clinical items: Emotional Abuse (EA), Physical Abuse (PA), Sexual Abuse (SA), Emotional Neglect (EN), and Physical Neglect (PN). Each subscale contains five items, and each item is rated using a 5-point Likert scale, ranging from 1 (never true) to 5 (very often true) (46). The total score ranges from 25 to 125. Higher scores represent more severe levels of childhood trauma. The CTQ-SF has been tested in Chinese populations and shown to be reliable and valid (47). The current study reports a Cronbach’s α = 0.915.

#### 2.2.4 Family functioning

Family functioning was evaluated using the General Functioning subscale of the McMaster Family Assessment Device (FAD-GF), originally developed by Epstein et al. in 1983 (48). This instrument contains 12 self-reported items rated on a four-point Likert scale (from “strongly agree” to “strongly disagree”). A higher total scores indicate worse family functioning (49). The FAD-GF has been widely utilized in clinical settings and empirical research, with multiple studies verifying its reliability and validity across diverse groups (50). In the current study, the internal consistency of this measure was satisfactory, with a Cronbach’s α of 0.887.

#### 2.2.5 Dysfunctional attitudes

The Dysfunctional Attitudes Scale (DAS) is a prevalent self-report instrument utilized to evaluate negative cognitive schemas regarded as cognitive vulnerability variables for depression (26). The most commonly used version, DAS Form A (DAS-A), contains 40 items graded on a 7-point Likert scale, with total scores ranging from 40 to 280; higher scores represent more severe dysfunctional beliefs (51). The Chinese version of DAS-A showed excellent psychometric properties (Cronbach’s α = 0.945; split-half reliability = 0.87) (52). In the current study, the internal consistency of this measure was satisfactory, with a Cronbach’s α of 0.924.

#### 2.2.6 Anhedonia

Anhedonia was assessed using the 14-item Snaith–Hamilton Pleasure Scale (SHAPS), which was developed by Snaith and Hamilton in 1995 (53). Each item is rated on a 4-point Likert scale (1 = “definitely agree” to 4 = “definitely disagree”), yielding a total score ranging from 14 to 56, with higher scores reflecting greater severity of anhedonia. The Chinese version of the SHAPS has been validated in both clinical and non-clinical populations, demonstrating strong internal consistency (Cronbach’s α = 0.90–0.91) (54). In the present study, the internal consistency of the SHAPS was also high (Cronbach’s α = 0.915).

### 2.3 Data analysis

All analyses were conducted using SPSS 26.0 (IBM Corp., Armonk, NY, USA) and Mplus 8.3 (Muthén & Muthén, Los Angeles, CA, USA). All tests were two-sided; *p* < 0.05 was considered statistically significant. Missing data (4.3%) were handled via multiple imputation under the missing at random (MAR) assumption, yielding five imputed datasets. Because the data violated normality, continuous variables in descriptive statistics were summarized as medians with interquartile ranges (IQRs), whereas categorical variables were summarized as frequencies and percentages. Bivariate associations among childhood maltreatment, family functioning, dysfunctional attitudes, anhedonia, and NSSI were examined using Spearman’s rank-order correlations. Mediation and moderated mediation models were estimated in Mplus 8.3 using robust maximum likelihood (MLR); for the binary outcome (NSSI), numerical integration via the Monte Carlo method was applied. As bootstrap confidence intervals are unavailable in this setting, the significance of indirect and interaction effects was evaluated using 95% confidence intervals from the Delta method; effects were deemed significant when the intervals excluded zero. All regression coefficients are reported as standardized estimates (β).

## 3 Results

### 3.1 Descriptive statistics and correlations

**Table 1** summarizes the demographic and clinical characteristics of the participants (n = 525). The sample consisted of 416 females (79.2%) and 109 males (20.8%), with a median age of 21 years (interquartile range, IQR = 19–22). A total of 61.5% of participants were experiencing their first episode of MDD. With respect to nonsuicidal self-injury (NSSI), 33.7% (n = 177) of participants reported a history of self-injury. Among these, nearly half (48.9%) engaged in NSSI at least once in the past year, followed by those who did so three times (21.1%), twice (19.3%), and four or more times (10.7%). Cutting was the most frequently reported method (49.3%), followed by stabbing/scratching (14.1%), punching/hitting objects (9.0%), hitting oneself (5.6%), and other methods (22.0%). Detailed demographic and clinical characteristics are presented in **Table 1**.

**Table 1:** Demographic and clinical characteristics of participants.

Characteristic	Median/Frequency	IQR/Percentage
Gender		
Female	416	79.2%
Male	109	20.8%
Age (years)	21	19-22
CTQ	41	34-54
FAD-GF	28	25-32
DAS	168	149-188
SHAPS	33	28-33
First-episode MDD,		
No	202	38.5%
Yes	323	61.5%
NSSI		
No	348	66.3%
Yes	177	33.7%
NSSI frequency		
1	87	48.9%
2	34	19.3%
3	37	21.1%
4	19	10.7%
NSSI method		
Cutting	87	49.3%
Stabbing/Scratching	25	14.1%
Punching/Hitting objects	16	9.0%
Hitting oneself	10	5.6%
Other	39	22.0%

N = 525. MDD, Major Depressive Disorder; CTQ, Childhood Trauma Questionnaire (higher scores indicate greater severity of childhood maltreatment). FAD-GF, Family Assessment Device-General Functioning subscale (higher scores indicate poorer family functioning). DAS, Dysfunctional Attitudes Scale (higher scores indicate more dysfunctional/rigid maladaptive beliefs). SHAPS, Snaith–Hamilton Pleasure Scale (higher scores indicate greater anhedonia). NSSI, nonsuicidal self-injury.

**Table 2** presents Spearman’s rank-order correlations among childhood maltreatment, family functioning, dysfunctional attitudes, anhedonia, and nonsuicidal self-injury (NSSI). The results indicate that childhood maltreatment was positively correlated with family functioning (r = 0.611, *p* < 0.001), dysfunctional attitudes (r = 0.207, *p* < 0.001), anhedonia (r = 0.197, *p* < 0.001), and NSSI (r = 0.106, *p* < 0.05). Family functioning showed significant correlations with dysfunctional attitudes (r = 0.203, *p* < 0.001) and anhedonia (r = 0.251, *p* < 0.001), but not with NSSI (r = 0.005, *p* > 0.05). Dysfunctional attitudes were positively associated with anhedonia (r = 0.319, *p* < 0.001) and NSSI (r = 0.164, *p* < 0.001). Anhedonia was not significantly related to NSSI (r = 0.072, *p* > 0.05). These findings provide preliminary support for the hypothesized associations and justify subsequent analyses of mediation and moderation. Collinearity diagnostics showed no evidence of multicollinearity (all tolerance > 0.2, all VIF < 5).

**Table 2:** Correlation analysis among childhood maltreatment, family functioning, dysfunctional attitudes, anhedonia, and NSSI.

Variable	Childhood maltreatment	Family functioning	Dysfunctional attitudes	Anhedonia	Nonsuicidal self-Injury
Childhood Maltreatment	1				
Family Functioning	0.611***	1			
Dysfunctional Attitudes	0.207***	0.203***	1		
Anhedonia	0.197***	0.251***	0.319***	1	
NSSI	0.106*	0.005	0.164***	0.072	1

All values represent Spearman correlation coefficients. n = 525. *P < 0.05, ***P < 0.01.

### 3.2 Mediation analysis

The mediation model, controlling for age, gender, and first-episode MDD status, showed a significant total effect of childhood maltreatment on NSSI (β = 0.101, *p* < 0.05, 95% CI [0.003, 0.199]) as well as a significant direct effect (β = 0.125, *p* < 0.05, 95% CI [0.010, 0.240]). The total indirect effect was not significant (β = −0.024, *p* = 0.514, 95% CI [−0.097, 0.049]). Examining the specific pathways, the indirect effect via family functioning was negative and nonsignificant (β = −0.060, *p* > 0.05, 95% CI [−0.130, 0.011]), whereas the pathway through dysfunctional attitudes was positive and significant (β = 0.035, *p* < 0.05, 95% CI [0.008, 0.063]). See **Table 3** for details.

**Table 3:** Mediation effects of family functioning and dysfunctional attitudes linking childhood maltreatment to nonsuicidal self-injury.

Effect	β	S.E.	Est./S.E	P-value	Bootstrap 95% CI (Lower)	Bootstrap 95% CI (Upper)
Total Effect	0.101	0.050	2.015	0.044	0.003	0.199
Direct Effect	0.125	0.059	2.133	0.033	0.010	0.240
Total indirect Effect	−0.024	0.037	−0.652	0.514	−0.097	0.049
CM→FF→NSSI	−0.060	0.036	−1.652	0.098	−0.130	0.011
CM→DA→NSSI	0.035	0.014	2.547	0.011	0.008	0.063

CM, childhood maltreatment; FF, family functioning; DA, dysfunctional attitudes; NSSI, nonsuicidal self-injury. All coefficients are standardized regression estimates.

### 3.3 Outcomes of the moderated mediation model

**Figure 2:**
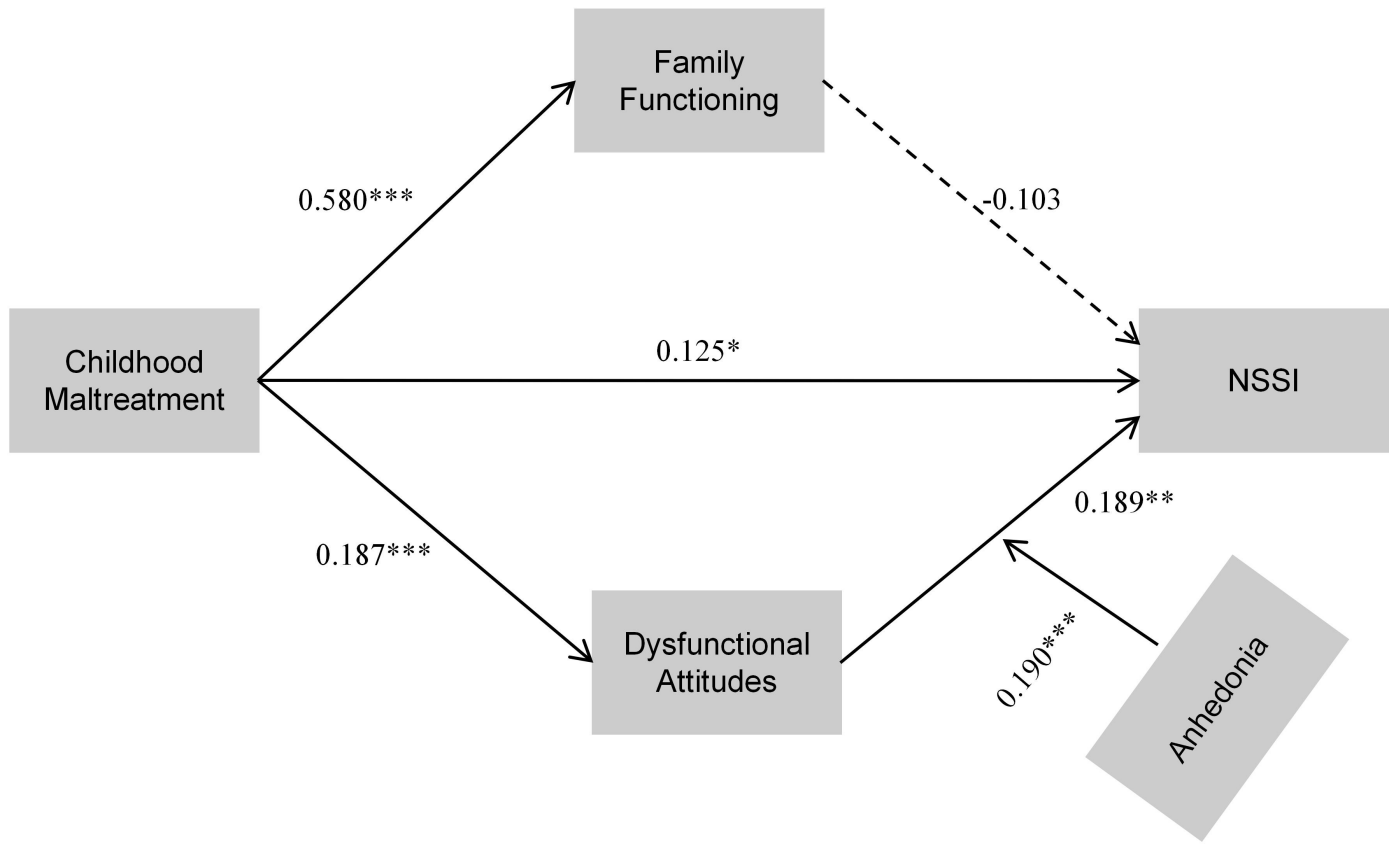
Standardized path diagram of the final moderated mediation model. Childhood maltreatment predicts NSSI via family functioning and dysfunctional attitudes, with anhedonia moderating the effect of dysfunctional attitudes on NSSI. *P < 0.05; **P < 0.01; ***P < 0.001.

After controlling for age, sex, and first-episode MDD status, a moderated mediation model revealed that dysfunctional attitudes significantly mediated the association between childhood maltreatment and nonsuicidal self-injury (NSSI), whereas family functioning did not exert a significant mediating effect. Moreover, anhedonia moderated the relationship between dysfunctional attitudes and NSSI. **Table 4** shows that the interaction between dysfunctional attitudes and anhedonia had a significant effect on NSSI (β = 0.190, *p* < 0.001, 95% CI [0.092, 0.288]).It means that dysfunctional attitudes were stronger at predicting NSSI in people with higher levels of anhedonia and weaker at predicting it in people with lower levels. **Figure 2** illustrates the overall moderated mediation model, including both the mediating and moderating effects.

**Table 4:** Moderated mediation effects of anhedonia on the associations between childhood maltreatment and NSSI.

Outcome variable	Predictor	β	S.E.	Est./S.E	P-value	Bootstrap 95% CI (Lower)	Bootstrap 95% CI (Upper)
FF	CM	0.580	0.029	20.174	0.000	0.524	0.637
DA	CM	0.187	0.042	4.452	0.000	0.105	0.269
NSSI	CM	0.125	0.059	2.133	0.033	0.010	0.240
	FF	−0.103	0.062	−1.665	0.096	−0.223	0.018
	DA	0.189	0.057	3.343	0.001	0.078	0.300
	Anhedonia	0.024	0.054	0.436	0.663	−0.083	0.130
	DA*Anhedonia	0.190	0.050	3.788	0.000	0.092	0.288

CM, childhood maltreatment; FF, family functioning; DA, dysfunctional attitudes; NSSI, nonsuicidal self-injury. All coefficients are standardized regression estimates.

**Figure 3:**
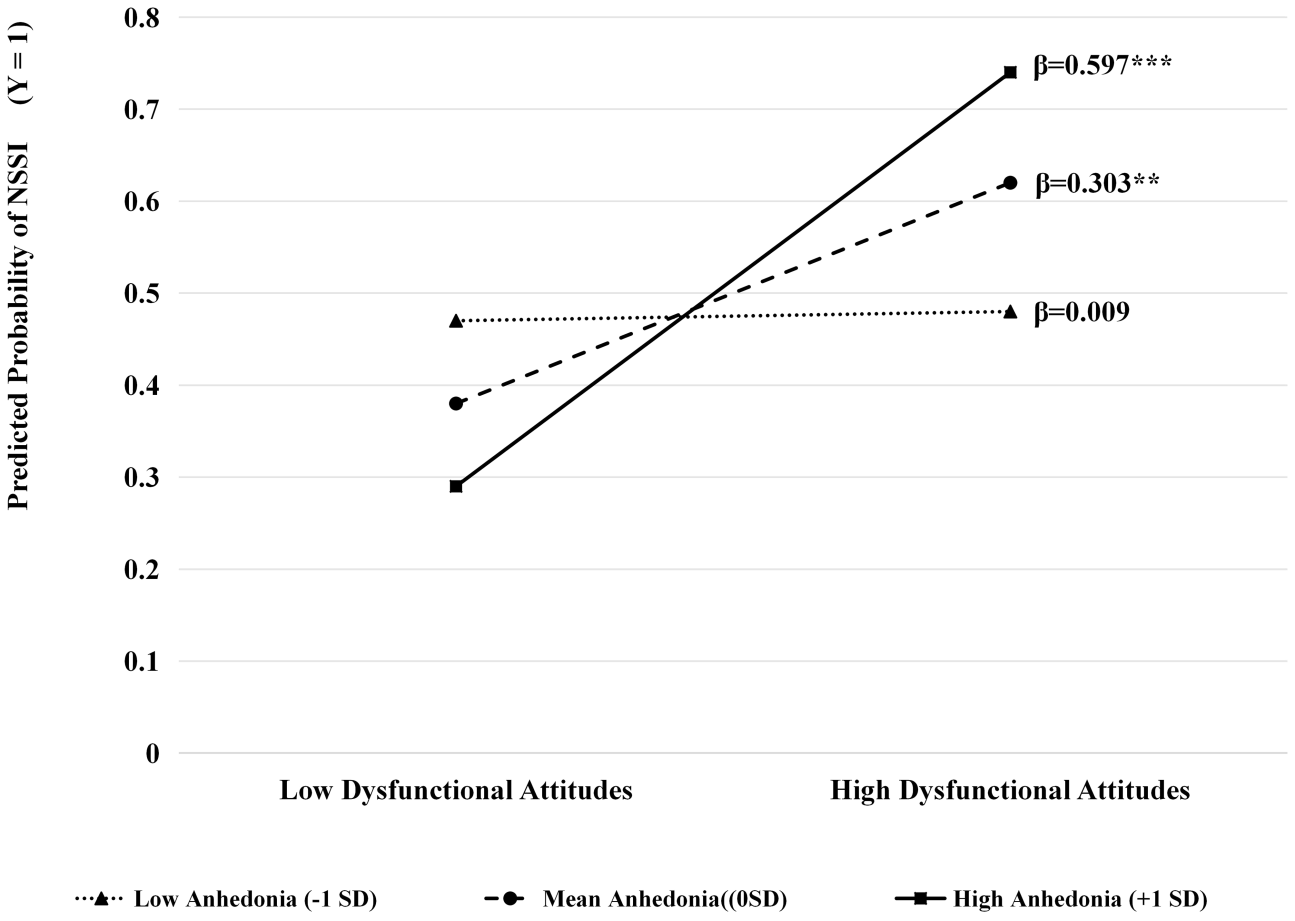
A simple slope plot illustrates the moderating effect of anhedonia on the relationship between dysfunctional attitudes and NSSI. **P < 0.01; ***P < 0.001.

Participants were categorized into high (+1 SD), average (0 SD), and low (–1 SD) anhedonia to investigate this interaction. Simple slope analysis (**Figure 3**) demonstrated a more pronounced positive slope for the high-anhedonia group (β = 0.597, *p* < 0.001), indicating that dysfunctional attitudes exerted a greater predictive influence on NSSI among depressed college students with higher levels of anhedonia. In contrast, the low-anhedonia group showed a relatively flat slope (β = 0.009, *p* > 0.05), suggesting a weaker or nonsignificant association between dysfunctional attitudes and NSSI in this cohort. At the mean level of anhedonia, the association remained significant (β = 0.303, *p* < 0.01), indicating a graded pattern of moderation instead of a threshold effect. **Figure 2** shows the overall moderated mediation model. It illustrates the standardized route coefficients for all pathways and emphasizes how anhedonia acts as a moderator.

## 4 Discussion

This study examined how childhood maltreatment contributes to NSSI among depressed college students by testing a moderated mediation model. The findings showed that childhood maltreatment predicted NSSI both directly and indirectly through dysfunctional attitudes, whereas the mediating role of family functioning was not supported. Moreover, anhedonia moderated the pathway from dysfunctional attitudes to NSSI, with stronger associations observed at higher levels of anhedonia. These findings make three contributions. First, they extend the cognitive vulnerability theory to a clinical sample of depressed college students, demonstrating its applicability beyond community populations. Second, they identify dysfunctional attitudes as a key cognitive mechanism linking childhood maltreatment to NSSI. Third, they highlight anhedonia as a boundary condition that intensifies the impact of maladaptive cognitions on self-injury. Together, the results clarify how early adverse experiences, cognitive vulnerability, and hedonic deficits jointly shape the risk of NSSI, offering implications for early identification and intervention.

### 4.1 The direct impact of childhood maltreatment on NSSI

In line with our hypothesis, childhood maltreatment has shown a substantial positive correlation with NSSI, confirming that early adverse experiences act as distal risk factors for self−injurious behavior (12, 55). Our finding supports the cognitive-behavioral vulnerability model (56). Individuals subjected to abuse or neglect during critical developmental phases may cultivate distorted self-schemas, emotional dysregulation, and maladaptive coping strategies, hence heightening their sensitivity to NSSI (57). Moreover, previous researches indicate that childhood maltreatment might impose persistent psychological effects through internalized mechanisms. Children who are subjected to abuse often lack the emotional and cognitive maturity to handle these experiences adequately (58). To maintain the attachment figure’s image as a source of security, individuals may internalize blame and embrace the abuser’s negative self-assessments (59). Over time, these internalized processes cultivate adverse implicit self-associations and cognitive vulnerabilities, elevating the risk of depression and self-directed violence, hence contributing to NSSI (60). These findings highlight the clinical significance of trauma-informed assessments in adolescent cohorts and indicate that, even in the absence of obvious symptoms, early maltreatment may lay the cognitive and emotional foundation for future self-injurious behavior. Future studies should further explore the complicated psychological mechanisms that connect childhood adversity to self−harming behaviors.

### 4.2 Mediation effects: dysfunctional attitudes and family functioning

The current study identified distinct mediation patterns connecting childhood maltreatment to NSSI. The findings confirmed Hypothesis 1, demonstrating that dysfunctional attitudes substantially mediated the relationship between childhood maltreatment and NSSI. This outcome corresponds with cognitive vulnerability theory. Individuals who experienced childhood maltreatment frequently develop cognitive distortions, heightened self-criticism, and inflexible perfectionistic beliefs (61). This background often leads them to see stressful situations negatively, exhibit increased sensitivity to failure, and form maladaptive cognitive patterns (62). Our findings support these conclusions, indicating that dysfunctional attitudes act as a proximal cognitive mechanism through which adverse childhood experiences increase the likelihood of NSSI. Maladaptive cognition is a notable risk factor for NSSI in individuals with MDD (63). Recent research indicates that college students with MDD and a history of childhood maltreatment are more likely to cultivate maladaptive attitudes and exhibit impaired emotional coping mechanisms (64). College students are particularly susceptible to multiple obstacles during the transition from adolescence to adulthood (65). It makes cognitive vulnerability especially important for this group.

Conversely, family functioning did not significantly mediate the relationship between childhood maltreatment and NSSI, thereby failing to support Hypothesis 2. Childhood maltreatment was a strong predictor of lower family functioning; however, this decrease in family functioning was not directly linked to NSSI in this group. One possible explanation is that during college years, students experience a decrease in emotional dependence on their parents, thereby making peers and romantic partners their primary sources of support. The literature on attachment transfer indicates that students may mitigate the negative effects of impaired family functioning by seeking compensatory support from their friends or romantic partners (66). Thus, the role of family functioning appears to be context-dependent, whereas dysfunctional attitudes constitute a more robust cognitive pathway. Interventions should therefore focus on identifying and modifying maladaptive cognitions, while also taking into account developmental stage and the influence of peer and romantic relationships when evaluating the impact of family functioning on NSSI.

### 4.3 Anhedonia as a stratified moderator between dysfunctional attitudes and NSSI

The present study demonstrated that anhedonia did not directly predict NSSI, but it significantly moderated the association between dysfunctional attitudes and NSSI. Specifically, the impact of dysfunctional attitudes on NSSI is notably stronger in individuals experiencing higher levels of anhedonia. At the same time, this relationship appears weaker or even nonsignificant in those with lower levels of anhedonia. These findings support Hypothesis 3, highlighting the significant moderating role of anhedonia in the cognitive pathway through which childhood maltreatment increases the risk of self-injurious behavior.

This result is broadly consistent with previous studies, which have shown that the direct association between anhedonia and NSSI is inconsistent; however, its role becomes evident when examined in interaction with cognitive or emotional vulnerabilities (67). By impairing reward processing and reducing positive affect, anhedonia diminishes individuals’ ability to buffer negative cognitions, thereby amplifying the behavioral consequences of dysfunctional attitudes (32). Furthermore, in the context of diminished ability to obtain pleasure from adaptive activities, self-injury may emerge as a more prominent option for immediate relief, so rewarding maladaptive behavior through negative reinforcement processes (68).

From a theoretical standpoint, these findings extend the Cognitive-Behavioral Vulnerability Model (36) by integrating hedonic deficits as a boundary condition that determines the extent to which maladaptive cognitions translate into self-injurious behavior. These findings suggest that anhedonia moderates the link between dysfunctional attitudes and NSSI, thereby clarifying the conditional nature of the cognitive pathway.

The results indicate that clinical therapies focused exclusively on altering maladaptive cognitions may prove inadequate. Effective prevention and treatment should concurrently address hedonic deficits through methods such as behavioral activation, positive affect enhancement, and reward-sensitivity training, while also continuing to address adverse cognitive patterns. Additionally, the concurrent evaluation of dysfunctional attitudes and anhedonia could be helpful in the identification of high-risk subgroups marked by simultaneous cognitive vulnerability and reward-processing deficiencies, hence facilitating more targeted and differentiated intervention options.

### 4.4 Limitations

Several limitations of this study need to be considered. First, the cross-sectional design prevents causal inference; longitudinal studies are necessary to establish the temporal ordering of childhood maltreatment, family functioning, dysfunctional attitudes, anhedonia, and NSSI. Second, all measurements depended on self-report, which can be influenced by recall bias and social desirability. Even though OSI items were used to assess presence, method, and frequency of NSSI, the function subscale was omitted. Moreover, due to the number of high-frequency cases was small, NSSI was dichotomized for analysis, which limited the capacity to assess severity adequately. Therefore, subsequent research needs to use comprehensive instruments that assess both frequency and functionality, and recruit larger and more diverse samples to allow reliable modeling of NSSI severity. Third, although demographic covariates were adjusted, important clinical variables such as depression severity, treatment status, and prior suicidal ideation or attempts were not available and hence not controlled. Future studies should integrate these variables to provide more precise estimates of the mechanisms linking childhood maltreatment and NSSI. Lastly, the sample consisted of college students with MDD recruited from hospitals and counseling centers, which may differ from community populations in severity and treatment-seeking tendencies. Future research should replicate findings in broader and more representative samples, and integrate additional psychosocial and biological factors to offer a more comprehensive understanding of the pathways between childhood maltreatment and NSSI.

## 5 Conclusion

This research improves our understanding of the relationship between childhood maltreatment and NSSI in college students with MDD by applying the cognitive-behavioral vulnerability model. Childhood maltreatment proved to be an effective predictor of NSSI, underscoring the persistent psychological effects of early adversity experiences. Contrary to our expectations, impaired family functioning—though related to childhood maltreatment—did not mediate NSSI risk, suggesting the presence of a suppression effect. It is plausible that emerging adults cope with dysfunctional family dynamics through emotional separation or by establishing external support systems, thereby weakening the influence of family dysfunction on self-injury.

Furthermore, anhedonia moderated the association between dysfunctional attitudes and NSSI in a crossover pattern: the effect of dysfunctional attitudes was significant in individuals with moderate to high levels of anhedonia, but not in those with low levels of anhedonia. This indicates that anhedonia amplifies the impact of cognitive vulnerability by undermining reward sensitivity and emotional resilience. From a clinical perspective, our findings highlight the effectiveness of customized interventions. Cognitive-behavioral therapy and schema-focused treatment might assist at-risk kids in restructuring dysfunctional beliefs. For individuals with significant anhedonia, behavioral activation treatments aimed at improving reward responsiveness may prove particularly advantageous. Since family functioning did not moderate the likelihood of NSSI, interventions for children who have experienced childhood maltreatment should extend beyond efforts to improve family relationships. A promising alternate option includes group-based therapies, like structured group counseling and peer support systems.

All of these observations show how important it is to have stratified, personalized interventions that deal with cognitive-affective weaknesses. Future studies should investigate variables, including peer interactions and neurobiological reward pathways, to inform more precise interventions.

## Data Availability

The raw data supporting the conclusions of this article will be made available by the authors, without undue reservation.

## References

[1] GBD. 2019 Mental Disorders Collaborators. Global, regional, and national burden of 12 mental disorders in 204 countries and territories, 1990-2019: a systematic analysis for the Global Burden of Disease Study 2019. Lancet Psychiatry. (2022) 9:137–50. doi: 10.1016/S2215-0366(21)00395-3, PMID: 35026139 PMC8776563

[2] GBD 2019 Diseases and Injuries Collaborators. Global burden of 369 diseases and injuries in 204 countries and territories, 1990–2019: a systematic analysis for the Global Burden of Disease Study 2019. Lancet. (2020) 396(10258):1204–22. doi: 10.1016/S0140-6736(20)30925-9, PMID: 33069326 PMC7567026

[3] Luo M Hao M Li X Liao J Wu C Wang Q. Prevalence of depressive tendencies among college students and the influence of attributional styles on depressive tendencies in the post-pandemic era. Front Public Health. (2024) 12:1326582. doi: 10.3389/fpubh.2024.1326582, PMID: 38333740 PMC10850216

[4] Nock MK. Self-injury. Annu Rev Clin Psychol. (2010) 6:339–63. doi: 10.1146/annurev.clinpsy.121208.131258, PMID: 20192787

[5] Klonsky ED Victor SE Saffer BY. Nonsuicidal self-injury: what we know, and what we need to know. Can J Psychiatry. (2014) 59:565–8. doi: 10.1177/070674371405901101, PMID: 25565471 PMC4244874

[6] Kiekens G Hasking P Claes L Boyes M Mortier P Auerbach RP. Predicting the incidence of non-suicidal self-injury in college students. Eur Psychiatry. (2019) 59:44–51. doi: 10.1016/j.eurpsy.2019.04.002, PMID: 31035219

[7] Kiekens G Hasking P Bruffaerts R Alonso J Auerbach RP Bantjes J. Non-suicidal self-injury among first-year college students and its association with mental disorders: Results from the World Mental Health International College Student (WMH-ICS) Initiative. psychol Med. (2021) 53:875. doi: 10.1017/S0033291721002245, PMID: 34140062 PMC8683565

[8] Qu D Wen X Liu B Zhang X He Y Chen D. Non-suicidal self-injury in Chinese population: a scoping review of prevalence, method, risk factors and preventive interventions. Lancet Regional Health – Western Pacific. (2023) 37:100794. doi: 10.1016/j.lanwpc.2023.100794, PMID: 37693882 PMC10485683

[9] Kang L Li R Liu H Ma S Sun S Zhang N. Nonsuicidal self-injury in undergraduate students with major depressive disorder: The role of psychosocial factors. J Affect Disord. (2021) 290:102–8. doi: 10.1016/j.jad.2021.04.083, PMID: 33993076

[10] Wilkinson P Kelvin R Roberts C Dubicka B Goodyer I. Clinical and psychosocial predictors of suicide attempts and nonsuicidal self-injury in the adolescent depression antidepressants and psychotherapy trial (ADAPT). AJP. (2011) 168:495–501. doi: 10.1176/appi.ajp.2010.10050718, PMID: 21285141

[11] Wu B Zhang H Chen J Chen J Liu Z Cheng Y. Potential mechanisms of non-suicidal self-injury (NSSI) in major depressive disorder: a systematic review. Gen Psychiatr. (2023) 36:e100946. doi: 10.1136/gpsych-2022-100946, PMID: 37655114 PMC10465892

[12] Liu RT Scopelliti KM Pittman SK Zamora AS. Childhood maltreatment and non-suicidal self-injury: a systematic review and meta-analysis. Lancet Psychiatry. (2018) 5:51–64. doi: 10.1016/S2215-0366(17)30469-8, PMID: 29196062 PMC5743605

[13] Wang W Wang X Duan G. Non-suicidal self-injury and suicidal ideation among Chinese college students of childhood emotional abuse: associations with rumination, experiential avoidance, and depression. Front Psychiatry. (2023) 14:1232884. doi: 10.3389/fpsyt.2023.1232884, PMID: 37588028 PMC10427149

[14] Brown RC Heines S Witt A Braehler E Fegert JM Harsch D. The impact of child maltreatment on non-suicidal self-injury: data from a representative sample of the general population. BMC Psychiatry. (2018) 18:181. doi: 10.1186/s12888-018-1754-3, PMID: 29884152 PMC5994090

[15] Wolff JC Thompson E Thomas SA Nesi J Bettis AH Ransford B. Emotion dysregulation and non-suicidal self-injury: A systematic review and meta-analysis. Eur Psychiatry. (2019) 59:25–36. doi: 10.1016/j.eurpsy.2019.03.004, PMID: 30986729 PMC6538442

[16] Calvo N Lugo-Marín J Oriol M Pérez-Galbarro C Restoy D Ramos-Quiroga J-A. Childhood maltreatment and non-suicidal self-injury in adolescent population: A systematic review and meta-analysis. Child Abuse Negl. (2024) 157:107048. doi: 10.1016/j.chiabu.2024.107048, PMID: 39332140

[17] Miller IW Ryan CE Keitner GI Bishop DS Epstein NB. “The McMaster Approach to Families: theory, assess- ment, treatment and research”. In: Walsh F, editor. Normal family processes 3rd Edn. New York, NY: Guilford Press. (2000). p. 561–583.

[18] Husky MM Lee S Sampson NA Borowski S Albor Y Alhadi AN. Childhood adversities and their associations with mental disorders in the World Mental Health International College Student surveys initiative. Psychiatry Res. (2025) 351:116585. doi: 10.1016/j.psychres.2025.116585, PMID: 40541041 PMC12291606

[19] Lippard ETC Nemeroff CB. The devastating clinical consequences of child abuse and neglect: increased disease vulnerability and poor treatment response in mood disorders. AJP. (2020) 177:20–36. doi: 10.1176/appi.ajp.2019.19010020, PMID: 31537091 PMC6939135

[20] Kong J Moorman SM Martire LM Almeida DM. The role of current family relationships in associations between childhood abuse and adult psychological functioning. J Gerontol B Psychol Sci Soc Sci. (2019) 74:858–68. doi: 10.1093/geronb/gby076, PMID: 29924362 PMC6566329

[21] Tomoda A Nishitani S Takiguchi S Fujisawa TX Sugiyama T Teicher MH. The neurobiological effects of childhood maltreatment on brain structure, function, and attachment. Eur Arch Psychiatry Clin Neurosci. (2024). doi: 10.1007/s00406-024-01779-y, PMID: 38466395 PMC12589315

[22] Li X Liu J Hu Y Huang X Li Y Li Y. The association of family functioning and suicide in children and adolescents: positive behavior recognition and non-suicidal self-injury as sequential mediators. Front Public Health. (2025) 13:1505960. doi: 10.3389/fpubh.2025.1505960, PMID: 40034164 PMC11873744

[23] Wang Y Luo B Hong B Yang M Zhao L Jia P. The relationship between family functioning and non-suicidal self-injury in adolescents: A structural equation modeling analysis. J Affect Disord. (2022) 309:193–200. doi: 10.1016/j.jad.2022.04.124, PMID: 35472474

[24] Zhou SC Zhou Z Tang Q Yu P Zou H Liu Q. Prediction of non-suicidal self-injury in adolescents at the family level using regression methods and machine learning. J Affect Disord. (2024) 352:67–75. doi: 10.1016/j.jad.2024.02.039, PMID: 38360362

[25] Cassels M van Harmelen A-L Neufeld S Goodyer I Jones PB Wilkinson P. Poor family functioning mediates the link between childhood adversity and adolescent nonsuicidal self-injury. J Child Psychol Psychiatry. (2018) 59:881–7. doi: 10.1111/jcpp.12866, PMID: 29363128 PMC6055861

[26] Weissman AN Beck AT. Development and validation of the dysfunctional attitude scale: A preliminary investigation (1978). Available online at: https://eric.ed.gov/?id=ED167619 (Accessed May 12, 2025).

[27] Strand ER Anyan F Hjemdal O Nordahl HM Nordahl H. Dysfunctional attitudes versus metacognitive beliefs as within-person predictors of depressive symptoms over time. Behav Ther. (2024) 55:801–12. doi: 10.1016/j.beth.2023.12.004, PMID: 38937051

[28] Yu T-F Liu L Shang L-N Xu F-F Chen Z-M Qian L-J. Dysfunctional attitudes, social support, negative life events, and depressive symptoms in Chinese adolescents: A moderated mediation model. World J Psychiatry. (2024) 14:1671–80. doi: 10.5498/wjp.v14.i11.1671, PMID: 39564176 PMC11572672

[29] Andersson H Aspeqvist E Dahlström Ö Svedin CG Jonsson LS Landberg Å. Emotional dysregulation and trauma symptoms mediate the relationship between childhood abuse and nonsuicidal self-injury in adolescents. Front Psychiatry. (2022) 13:897081. doi: 10.3389/fpsyt.2022.897081, PMID: 35966492 PMC9366744

[30] Snaith P. Anhedonia: exclusion from the pleasure dome. BMJ. (1992) 305:134–4. doi: 10.1136/bmj.305.6846.134, PMID: 1515824 PMC1883239

[31] RosenbergBMYoungKSNusslockRZinbargRECraskeMG. Anhedonia is associated with overgeneralization of conditioned fear during late adolescence and early adulthood. J Anxiety Disord. (2024) 105:102880. doi: 10.1016/j.janxdis.2024.102880, PMID: 38833961

[32] Liu W Roiser JP Wang L Zhu Y Huang J Neumann DL. Anhedonia is associated with blunted reward sensitivity in first-degree relatives of patients with major depression. J Affect Disord. (2016) 190:640–8. doi: 10.1016/j.jad.2015.10.050, PMID: 26590511 PMC5330646

[33] Auerbach RP Pagliaccio D Pizzagalli DA. Towards an improved understanding of anhedonia in youth. JAMA Psychiatry. (2019) 76:571–3. doi: 10.1001/jamapsychiatry.2018.4600, PMID: 30865251 PMC6817369

[34] Yang X Wang D Liu S Liu G Harrison P. Stress and suicidal ideation: the role of state or trait anhedonia in a moderated mediation model. Suicide Life Threat Behav. (2020) 50:502–14. doi: 10.1111/sltb.12605, PMID: 31750566

[35] Rzepa E McCabe C. Dimensional anhedonia and the adolescent brain: reward and aversion anticipation, effort and consummation. BJPsych Open. (2019) 5:e99. doi: 10.1192/bjo.2019.68, PMID: 31724528 PMC6949536

[36] Reilly LC Ciesla JA Felton JW Weitlauf AS Anderson NL. Cognitive vulnerability to depression: A comparison of the weakest link, keystone and additive models. Cogn Emotion. (2012) 26:521–33. doi: 10.1080/02699931.2011.595776, PMID: 21851251 PMC4083570

[37] Guerry JD Prinstein MJ. Longitudinal prediction of adolescent nonsuicidal self-injury: examination of a cognitive vulnerability-stress model. J Clin Child Adolesc Psychol. (2009) 39:77–89. doi: 10.1080/15374410903401195, PMID: 20390800 PMC4626882

[38] van de Leemput IA Wichers M Cramer AOJ Borsboom D Tuerlinckx F Kuppens P. Critical slowing down as early warning for the onset and termination of depression. Proc Natl Acad Sci U.S.A. (2014) 111:87–92. doi: 10.1073/pnas.1312114110, PMID: 24324144 PMC3890822

[39] Battle DE. Diagnostic and statistical manual of mental disorders (DSM). Codas. (2013) 25:191–2. doi: 10.1590/s2317-17822013000200017, PMID: 24413388

[40] Amorim P Lecrubier Y Weiller E Hergueta T Sheehan D. DSM-IH-R Psychotic Disorders: procedural validity of the Mini International Neuropsychiatric Interview (MINI). Concordance and causes for discordance with the CIDI. Eur Psychiatry. (1998) 13:26–34. doi: 10.1016/S0924-9338(97)86748-X, PMID: 19698595

[41] Martin J Cloutier PF Levesque C Bureau J-F Lafontaine M-F Nixon MK. Psychometric properties of the functions and addictive features scales of the Ottawa Self-Injury Inventory: A preliminary investigation using a university sample. psychol Assess. (2013) 25:1013–8. doi: 10.1037/a0032575, PMID: 23647037

[42] Guérin-Marion C Martin J Deneault A-A Lafontaine M-F Bureau J-F. The functions and addictive features of non-suicidal self-injury: A confirmatory factor analysis of the Ottawa self-injury inventory in a university sample. Psychiatry Res. (2018) 264:316–21. doi: 10.1016/j.psychres.2018.04.019, PMID: 29665561

[43] Chen S Cui X Liao M Zhang F Ren Y Cao J. Comprehensive analysis of key genes and biological pathways after tibial cortex transverse transport surgery in diabetic foot ulcers. Int J Mol Sci. (2025) 26:9862. doi: 10.3390/ijms26189862

[44] Zhang ZX Huang XX Hu J Xue YN Jia LY Tao FB. Association of interaction between mental health literacy and non-suicidal self-injury with suicidal behaviors among middle school students: a cross-sectional survey in three China cities. Chin J Public Health. (2022) 38:1517–22. doi: 10.11847/zgggws1138596

[45] Bernstein DP Fink L Handelsman L Foote J Lovejoy M Wenzel K. Initial reliability and validity of a new retrospective measure of child abuse and neglect. Am J Psychiatry. (1994) 151:1132–6. doi: 10.1176/ajp.151.8.1132, PMID: 8037246

[46] Bernstein DP Stein JA Newcomb MD Walker E Pogge D Ahluvalia T. Development and validation of a brief screening version of the Childhood Trauma Questionnaire. Child Abuse Negl. (2003) 27:169–90. doi: 10.1016/s0145-2134(02)00541-0, PMID: 12615092

[47] Jiang W-J Zhong B-L Liu L-Z Zhou Y-J Hu X-H Li Y. Reliability and validity of the Chinese version of the Childhood Trauma Questionnaire-Short Form for inpatients with schizophrenia. PloS One. (2018) 13:e0208779. doi: 10.1371/journal.pone.0208779, PMID: 30543649 PMC6292582

[48] Epstein NB Baldwin LM Bishop DS. The mcMaster family assessment device. J Marital Family Ther. (1983) 9:171–80. doi: 10.1111/j.1752-0606.1983.tb01497.x

[49] Miller IW Epstein NB Bishop DS Keitner GI. THE mcMASTER FAMILY ASSESSMENT DEVICE: RELIABILITY AND VALIDITY*. J Marital Family Ther. (1985) 11:345–56. doi: 10.1111/j.1752-0606.1985.tb00028.x

[50] Cong CW Tan SA Nainee S Tan C-S. Psychometric qualities of the mcMaster family assessment device–general functioning subscale for Malaysian samples. Int J Environ Res Public Health. (2022) 19:2440. doi: 10.3390/ijerph19042440, PMID: 35206628 PMC8875097

[51] de Graaf LE Roelofs J Huibers MJH. Measuring dysfunctional attitudes in the general population: the dysfunctional attitude scale (form A) revised. Cogn Ther Res. (2009) 33:345–55. doi: 10.1007/s10608-009-9229-y, PMID: 19623267 PMC2712063

[52] Wong DFK Chan KS Lau Y. The reliability and validity of the chinese version of the dysfunctional attitudes scale form a (Das-A) in a community sample. Int J Psychiatry Med. (2008) 38:141–52. doi: 10.2190/PM.38.2.b, PMID: 18724566

[53] Snaith RP Hamilton M Morley S Humayan A Hargreaves D Trigwell P. A scale for the assessment of hedonic tone the Snaith-Hamilton Pleasure Scale. Br J Psychiatry. (1995) 167:99–103. doi: 10.1192/bjp.167.1.99, PMID: 7551619

[54] Zhang P Zhang N Fang S He J Fan L Luo X. Factor Structure and Measurement Invariance of the Chinese version of the Snaith-Hamilton Pleasure Scale (SHAPS) in Non-clinical and Clinical populations. J Affect Disord. (2021) 281:759–66. doi: 10.1016/j.jad.2020.11.068, PMID: 33229024

[55] Fu W Li X Ji S Yang T Chen L Guo Y. The relationship between childhood trauma and non-suicidal self-injury behavior in adolescents with depression: the mediating role of rumination. Psychol Res Behav Manag. (2024) 17:1477–85. doi: 10.2147/PRBM.S448248, PMID: 38606089 PMC11007121

[56] Abramson LY Alloy LB Hankin BL Haeffel GJ MacCoon DG Gibb BE. “Cognitive vulnerability-stress models of depression in a self-regulatory and psychobiological context”. In: Handbook of depression. New York, NY, US: The Guilford Press (2002). (2002). p. 268–94. doi: 10.1097/00005053-200301000-00022

[57] Yang L Du X Huang M. Childhood maltreatment and non-suicidal self-injury: the mediating role of mentalization and depression. Eur J Psychotraumatol. (2025) 16:2466279. doi: 10.1080/20008066.2025.2466279, PMID: 39995338 PMC11864010

[58] Zheng T Zhou H Ni Z Zou L Yan C. Childhood trauma impacts internalizing problems in early adulthood: the affective implications of olfactory dysfunctions. Humanit Soc Sci Commun. (2024) 11:1395. doi: 10.1057/s41599-024-03933-2

[59] Lahousen T Unterrainer HF Kapfhammer H-P. Psychobiology of attachment and trauma-some general remarks from a clinical perspective. Front Psychiatry. (2019) 10:914. doi: 10.3389/fpsyt.2019.00914, PMID: 31920761 PMC6920243

[60] You J Ren Y Zhang X Wu Z Xu S Lin M-P. Emotional dysregulation and nonsuicidal self-injury: A meta-analytic review. Neuropsychiatry. (2018) 8(6):733–48. doi: 10.4172/Neuropsychiatry.1000399

[61] Üçok A Noyan H Gülöksüz S Saka MC Alptekin K Atbaşoğlu C. The relationship between childhood trauma, psychotic symptoms, and cognitive schemas in patients with schizophrenia, their siblings, and healthy controls: results from the EU-GEI study. Psychol Med. (2024) 54:2414–25. doi: 10.1017/S0033291724000540, PMID: 38606591

[62] McLaughlin KA Lambert HK. Child trauma exposure and psychopathology: mechanisms of risk and resilience. Curr Opin Psychol. (2017) 14:29–34. doi: 10.1016/j.copsyc.2016.10.004, PMID: 27868085 PMC5111863

[63] Palmer-Cooper EC Woods C Richardson T. The relationship between dysfunctional attitudes, maladaptive perfectionism, metacognition and symptoms of mania and depression in bipolar disorder: The role of self-compassion as a mediating factor. J Affect Disord. (2023) 341:265–74. doi: 10.1016/j.jad.2023.08.117, PMID: 37633530

[64] Jugessur R Zhang Y Qin X Wang M Lu X Sun J. Childhood maltreatment predicts specific types of dysfunctional attitudes in participants with and without depression. Front Psychiatry. (2021) 12:728280. doi: 10.3389/fpsyt.2021.728280, PMID: 34744822 PMC8568793

[65] Pedrelli P Nyer M Yeung A Zulauf C Wilens T. College students: mental health problems and treatment considerations. Acad Psychiatry. (2015) 39:503–11. doi: 10.1007/s40596-014-0205-9, PMID: 25142250 PMC4527955

[66] Gillath O Karantzas GC Fraley RC. Adult attachment: A concise introduction to theory and research. San Diego, CA: Elsevier Academic Press (2016). doi: 10.1016/C2013-0-09705-8

[67] Zielinski MJ Veilleux JC Winer ES Nadorff MR. A short-term longitudinal examination of the relations between depression, anhedonia, and self-injurious thoughts and behaviors in adults with a history of self-injury. Compr Psychiatry. (2017) 73:187–95. doi: 10.1016/j.comppsych.2016.11.013, PMID: 28040576 PMC5458327

[68] Antezana L Garcia KM Carlton CN Villalongo Andino M Gatto AJ Richey JA. Transdiagnostic correlates of nonsuicidal self-injury: the roles of anhedonia, repetitive negative thinking, and trait mindfulness. J Psychopathol Behav Assess. (2024) 46:566–79. doi: 10.1007/s10862-024-10130-7, PMID: 40756307 PMC12316051

